# Forensic genetics and genomics: Much more than just a human affair

**DOI:** 10.1371/journal.pgen.1006960

**Published:** 2017-09-21

**Authors:** Miguel Arenas, Filipe Pereira, Manuela Oliveira, Nadia Pinto, Alexandra M. Lopes, Veronica Gomes, Angel Carracedo, Antonio Amorim

**Affiliations:** 1 Department of Biochemistry, Genetics and Immunology, University of Vigo, Vigo, Spain; 2 Instituto de Investigação e Inovação em Saúde (i3S), University of Porto, Porto, Portugal; 3 Institute of Molecular Pathology and Immunology of the University of Porto (IPATIMUP), Porto, Portugal; 4 Interdisciplinary Centre of Marine and Environmental Research (CIIMAR), University of Porto, Porto, Portugal; 5 Faculty of Sciences, University of Porto, Porto, Portugal; 6 Centre of Mathematics of the University of Porto, Porto, Portugal; 7 Institute of Forensic Sciences Luis Concheiro, University of Santiago de Compostela, Santiago de Compostela, Spain; 8 Genomics Medicine Group, CIBERER, University of Santiago de Compostela, Santiago de Compostela, Spain; George Washington University, UNITED STATES

## Abstract

While traditional forensic genetics has been oriented towards using human DNA in criminal investigation and civil court cases, it currently presents a much wider application range, including not only legal situations sensu stricto but also and, increasingly often, to preemptively avoid judicial processes. Despite some difficulties, current forensic genetics is progressively incorporating the analysis of nonhuman genetic material to a greater extent. The analysis of this material—including other animal species, plants, or microorganisms—is now broadly used, providing ancillary evidence in criminalistics in cases such as animal attacks, trafficking of species, bioterrorism and biocrimes, and identification of fraudulent food composition, among many others. Here, we explore how nonhuman forensic genetics is being revolutionized by the increasing variety of genetic markers, the establishment of faster, less error-burdened and cheaper sequencing technologies, and the emergence and improvement of models, methods, and bioinformatics facilities.

## Introduction

Forensic genetics derives from a late offshoot of the big tree resulting from the conjunction between legal medicine and criminalistics (for the distinction between forensic genetics and other forensic sciences, see [1–3]). Its historical evolution shows substantial theoretical and technological developments and has, meanwhile, turned this discipline into a broad and independent scientific area for which it is becoming more and more difficult to identify its most remote ancestors. The evolution of modern societies substantially broadened the forensic framework by introducing new forms of resolution of disputes, allowing space for prevention, and regulating more restrictively the prosecution investigations. This means that a potentially forensic situation is the one for which 2 or more sides (individual persons or institutions) agree on the reality of the facts but do disagree on the causes or authorship (thereafter, the term “forensic” is used for these scenarios). Thus, civil litigations (and not just criminal) are common but also conflicts (which are increasing with time) that are attempted to be solved outside a formal court environment [4].

**Fig 1:**
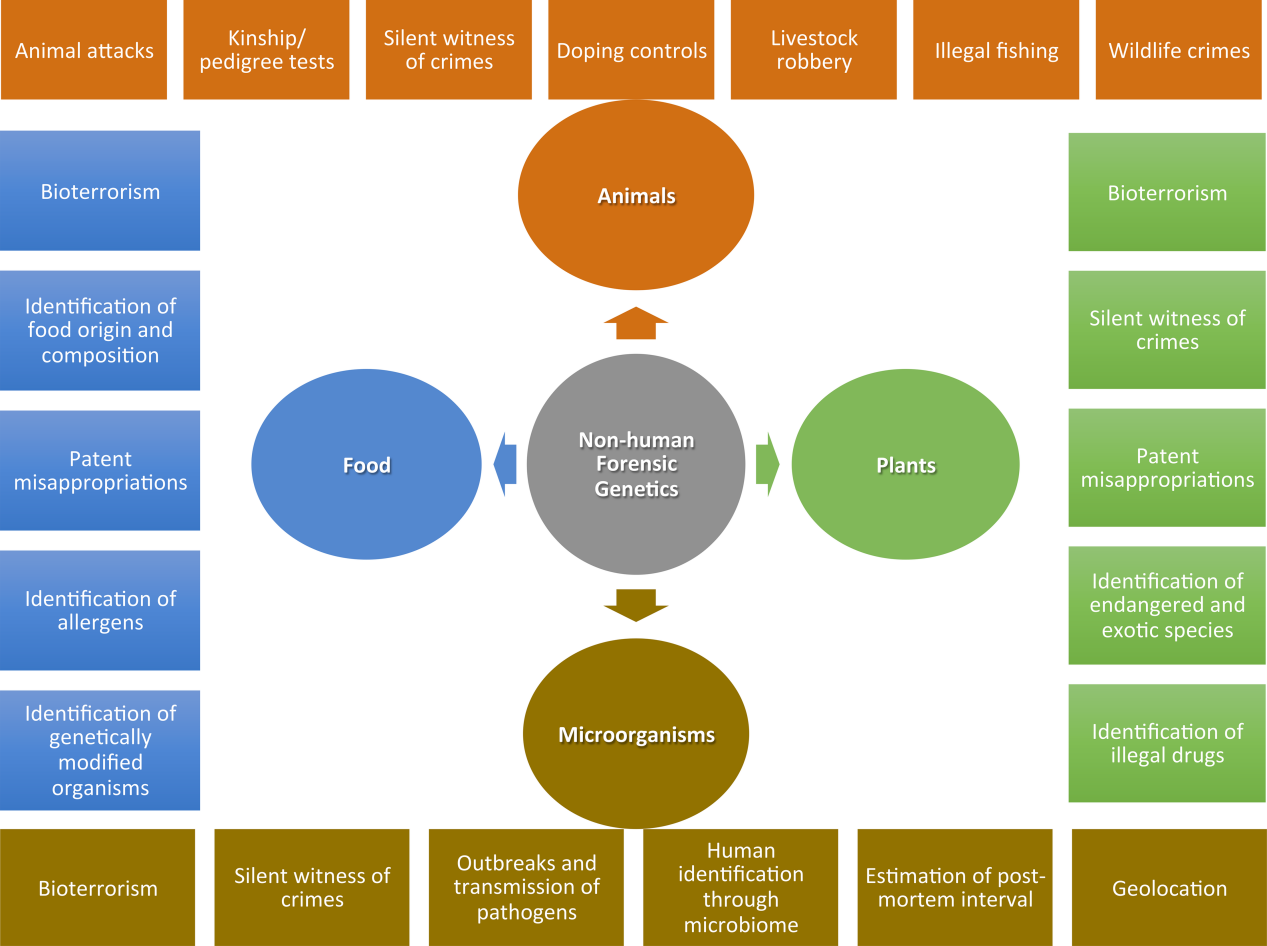
Most relevant applications of the zoology, botany, microbiology, and food analysis and traceability sciences to NHFG. Diverse examples for each of these applications are shown in S1 Table and S2 Table (see also S1 Text) and described in the section applications of NHFG. NHFG, nonhuman forensic genetics.

It is surprising that most of the life span of the discipline has been devoted to human genetics [e.g., 5], since a number of disagreements on questions intrinsically related to nonhuman materials always existed and, even when strictly human issues are at stake (such as the identification of a murderer), evidence from nonhuman sources can be crucial or are just the sole type of available evidence [e.g., 6]. This has been recognized by the first scientific journal explicitly devoted to forensic genetics (*Forensic Science International*: *Genetics*), when defining it as “The application of genetics to human and nonhuman material (in the sense of a science with the purpose of studying inherited characteristics for the analysis of inter- and intraspecific variations in populations) for the resolution of legal conflicts” [7]. Consequently, the division between human and nonhuman forensic genetics (HFG and NHFG, respectively) is not just the result of an anthropocentric historical tradition; rather, it could be derived from the different genomic architectures of the involved organisms [8]. Importantly, a number of forensically relevant questions are unthinkable in purely human terms (Fig 1), and in this review, we highlight their relevance.

Below, we begin by describing the commonly used methodologies, including genotyping and sequencing strategies, evolutionary frameworks, and statistical approaches. Next, we broadly describe applications of NHFG based on diverse biological sources. Finally, we discuss the future of the discipline, including needs and recommendations.

## Experimental methodologies in NHFG

**Fig 2:**
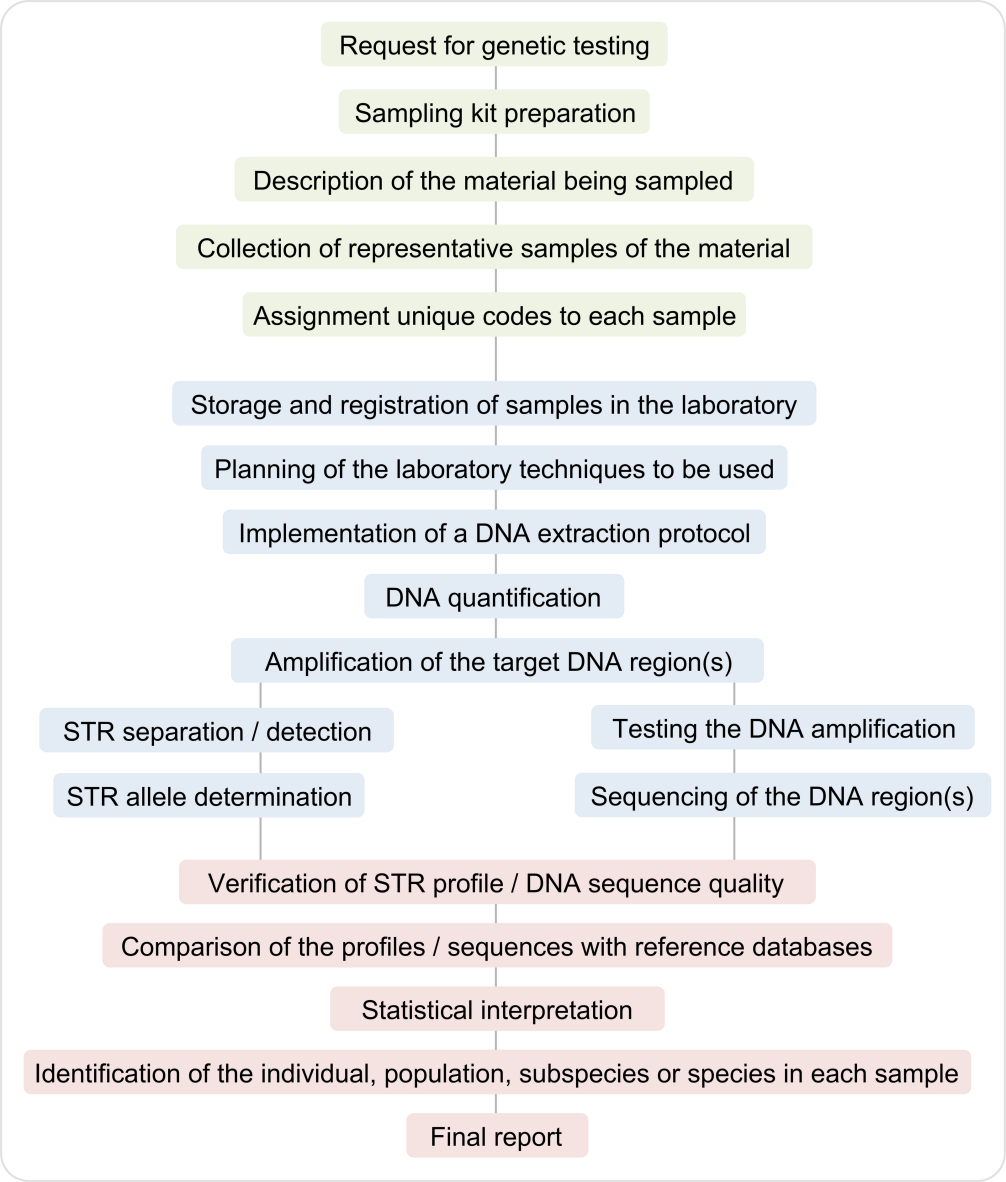
Pipeline showing the main steps usually involved in processing forensic nonhuman DNA samples. The exact procedure will depend on the conditions available at each laboratory. The process starts with the evaluation of the case and sample collection (green boxes). The procedure continues in the laboratory, where the DNA is extracted from the biological source material and analyzed according to an appropriate protocol (blue boxes). The genetic information is then compared with reference databases and the results are described in a written report (red boxes).

The techniques of forensic genetics originally developed for humans were rapidly adapted to other sources of genetic material. The experimental pipeline used in NHFG (Fig 2) starts with a request for a genetic testing. Next, samples are collected using a sampling kit (either commercial or assembled in the laboratory) and transported to the laboratory under proper conditions. An accurate description of the biological nature of the sample is usually included, and a unique code must be assigned to each collected sample. If the request is part of a legal procedure, not only traceability but also the strict maintenance of the chain of custody (chronological documentation of the evidence) are key issues. The procedure continues in the laboratory, where the genetic material is extracted from the samples using an appropriate and validated protocol. However, certain urgent situations (e.g., bioterrorism) may require the use of methods that were not previously validated. The laboratory may have to deal with new kinds of biological material or taxonomic groups never studied before. In such cases, the laboratory has to be able to develop a valid strategy to extract DNA with sufficient quality and quantity for downstream analyses.

Care must be taken when extracting and storing the genetic material to maintain integrity. Storage of nonhuman evidence does not create any specific problem (except required space) that is not common to HFG, and it must be handled in the same way as human material, following the same rules in labelling, chain of custody recording, etc. However, reproducibility in NHFG is clearly a major issue, especially when dealing with wildlife and environmental materials, due to inherent sampling difficulties. Therefore, validation studies cannot be performed in the same strict sense as they are in HFG (for a guideline on these problems, see [9]).

The selection of the genetic test depends on the question to be addressed (see next subsection). For instance, the sequencing of a PCR-amplified genetic region (e.g., cytochrome b [CYTB], cytochrome c oxidase I [COI], and ribosomal RNA [rRNA] genes) is often used for species identification. The identification of individuals can be achieved using a number of markers sufficient to provide high power of discrimination. In any case, it is important to check the quality of profiles or DNA sequences before analysing the results. The genetic information obtained is then compared with other genetic information (i.e., derived from reliable databases [e.g., 10] or reference sample[s]) considering statistical analyses. The experimental workflow ends with a report describing the technical procedures applied and the answers to the question(s) of the request.

## Genotyping and sequencing

Genetic identification is based on polymorphic DNA markers that can provide sufficient discriminatory resolution. Traditionally, PCR-based methodologies designed to generate short amplicons, such as Rapid Amplification of Random DNA (RAPD) [11], Inter Simple Sequence Repeats (ISSR) [12], and Amplified Fragment Length Polymorphism (AFLP) [13], were applied in NHFG analyses. Two relevant forensic cases applying RADP are the analysis of plant (seed pods) DNA in a murder case in Phoenix [14], and the analysis of the outbreak of human anthrax occurred in Sverdlovsk (Ekaterinburg, Russia) [15].

However, due to their limitations, these techniques were rapidly replaced by Simple Sequence Repeats (SSRs, Short Tandem Repeats [STRs], or microsatellites) and Single Nucleotide Polymorphisms (SNPs) [e.g., 16]. The development of reduced-size STR amplicons (miniSTRs) can provide easy PCR amplification of degraded DNA samples, better estimation of mutation rates and allele frequencies, and construction of allelic ladders for accurate classification of alleles. Alternative methods to PCR include technologies such as nucleic acid sequence-based amplification (NASBA) and loop-mediated isothermal amplification (LAMP) [17, 18]. Later, a more advanced post-PCR technique, high-resolution DNA melting (HRM) analysis, which is based on the detection of small differences in amplicon melting (dissociation) curves, was also considered for NHFG [e.g., 19]. On the other hand, for DNA barcoding, a technique widely used in species identification [e.g., 20], is necessary to determine the DNA base composition by targeting specific regions with the Sanger sequencing method. Recently, the arrival of next generation sequencing (NGS) has also revolutionized forensic genetics [21]. These new technologies provide clear advantages regarding high-throughput due to an extensive multiplexing capacity and parallel sequencing of millions of molecules (Multiple Parallel Sequencing, MPS), allowing a faster and more informative analysis (i.e., characterization of allelic and copy-number variation, CNV) of the genomic material in a sample. Concerning NHFG, MPS is particularly useful for the analysis of samples of complex mixtures since untargeted approaches can be used without prior knowledge about the source. MPS presents additional advantages for NHFG such as the detection of rare polymorphisms, high resolution of genetic analysis, and informative power.

## Methodologies for the evaluation of statistical evidence and for evolutionary analysis

Advances in NHFG are also caused by the progress of bioinformatics and statistical tools. A clear example is the emergence and evolution of the bioinformatics pipeline for the assembly of reads generated in MPS [see for a review, 22]. We next describe 2 analytic facets of crucial importance for NHFG: the quantitative evaluation of DNA evidence in the context of identification kinship and population/species assignment (i.e., in shallow evolutionary timescales) and the evolutionary analysis of genetic data (e.g., in transmission of fast evolving pathogens).

## Statistical evaluation of evidence

Here, we overview the use of nonhuman genetic material (NHGM) as ancillary evidence to solve classical forensic problems and in cases that fall outside the civil and criminal human authorship or responsibility.

### NHGM used as auxiliary evidence in litigations of human authorship

NHGM can play a crucial role in the investigation of diverse criminal disputes with the aim of identifying the human individual(s) who committed a crime or is/are responsible for some liability or damage. Historically, the first contributions correspond to situations in which NHGM is used as a silent witness resulting from involuntary transfer and leading to the so-called transfer or associative evidence. This type of NHGM usage is best illustrated in criminalistics where it is increasingly important, as perpetrators are progressively avoiding carefully leaving their biological traces in the crime scene. However, they can, for example, inadvertently leave their pets’ hairs at the crime scene or, inversely, to carry the victims’ pet biological material [e.g., 6]. Although pets are exceedingly common in modern households, many more exotic situations fit with this silent witness type of NHGM use (i.e., knotgrass [23], mosses [24], oak [25], and soil DNA [26]; see S1 Table and section applications of NHFG). Besides the existing variety of applications, new developments are already at sight such as the genetic profiling of microbiomes and microbial metagenomics [e.g., 27, 28–30], or in a not too distant future, the identification of transmitted strains of pathogens or commensals even many years after the crime (as it has been already done for viral transmissions [e.g., 31, 32–36]).

### NHGM used as evidence in other litigations

There are several cases for which the expert evidence is used to deal with a law/regulation infringement, irrespectively of the human authorship or responsibility (which may be investigated separately). Here, a comprehensive classification is complex, given the dynamic evolution of the applications and the diversity of ever-growing fields for which laws are constantly being issued. Nevertheless, we must note a clear difference between the applications in which the litigation treats the nonhuman in a framework similar to human cases (individualization and kinship) and a plethora of other applications.

Concerning the former, both theoretical frameworks and technological platforms developed for humans can be almost directly translated. This category includes several scenarios: (*i*) an individual living being (e.g., an animal) is the direct causation of an injury to another living being or causes property damages [e.g., 37, 38]; (*ii*) the genetic relationship (e.g., paternity) of a living being to another one is unsettled [e.g., 39, 40]; (*iii*) the identity of the donor of a sample is under dispute (e.g., doping controls in horse races) [e.g., 41] (S2 Table).

As for the latter, a wide range of examples considering NHGM as auxiliary or direct evidence in litigations are derived from diverse subdisciplines such as forensics zoology, botany, microbiology, and food analysis (see Fig 1 and section applications of NHFG).

In any case, statistical analysis should provide likelihoods of observations, rather than categorical answers, and at least 2 alternative, mutually exclusive hypotheses should be formulated. Broadly speaking, statistical evaluation in NHFG can be required for 3 major scenarios:

1. Individual identification or kinship. It involves cases such as “was a given dog the perpetrator of the attack?” or “is a given foal the offspring of a given highly prized horse?”
2. Species identification. It involves interspecies cases such as “does the label of a processed fish product agree with the species of origin?”
3. Subspecific assignment/identification. It involves cases related with breed, variety, or populations such as “was the attack perpetrated by a dog or by a wolf?”

Regarding scenario A, similarly to what has been established for humans, autosomal STRs are preferentially considered and analysed with a Bayesian approach (in which prior odds are combined with probabilities of the genotypic observations assuming the alternative hypotheses). Nevertheless, several difficulties can arise in practice, especially when dealing with small sized and/or poorly studied populations, as in endangered species. The lack of knowledge in the population structure and sampling errors obviously has a serious impact on the confidence of the parameter estimates. The software developed in HFG for kinship and identification can be used in this scenario. For instance, the computer programs GDA [42] and GenePop [43] can be applied to test Hardy-Weinberg Equilibrium and to estimate population genetics parameters, while the program Familias [44] can be used to compute kinship likelihood ratios.

Regarding scenario B, the traditional procedure consists of comparing sequences that are highly variable among species but highly conserved within species, in the so-called DNA barcoding [45]. An alternative approach relies on comparing lengths of insertion and deletion polymorphisms without requiring DNA sequencing [46, 47]. Importantly, these approaches are only possible due to the existence and maintenance of reliable and public databases such as GenBank, EMBL, and Bold. Note that the increasing number and length of sequences existing in databases and the development of automated mechanisms to prevent misclassified sequences would allow more confidence in species identification. Finally, the statistical significance of sequence comparison should be computed [48] and reported.

Regarding scenario C, the selection of the genetic marker depends on the investigated species. For metazoan, the most used markers are regions of the mitochondrial genome (and plastid for Plantae) that can provide accurate distinction between subspecies [e.g., 49] and autosomal regions of nuclear DNA [e.g., 50]. The program STRUCTURE [51] is widely used for the Bayesian assignment of an individual to a population (or subspecies). Again, the statistical evaluation should be performed and reported.

## Evolutionary analysis of genetic data in forensic genetics

Diverse organisms involved in forensic studies present short generation times and belong to large populations (as most pathogens). Therefore, relationships between queried and control samples are usually obtained under the light of evolutionary analyses since those samples most likely belong to distant generations [52]. The evolutionary analysis not only provides the identification of genetic relationships (dealing with questions like, “is the suspect the cause of the studied transmission or outbreak?” or “which individuals were infected by the suspect and which individuals were infected or coinfected from other sources?”) [e.g., 31–36, 53, 54, 55], but also allows the estimation of the timing of transmission events (i.e., infection date of each individual, including the individual that generated the outbreak) [e.g., 53, 54].

**Fig 3:**
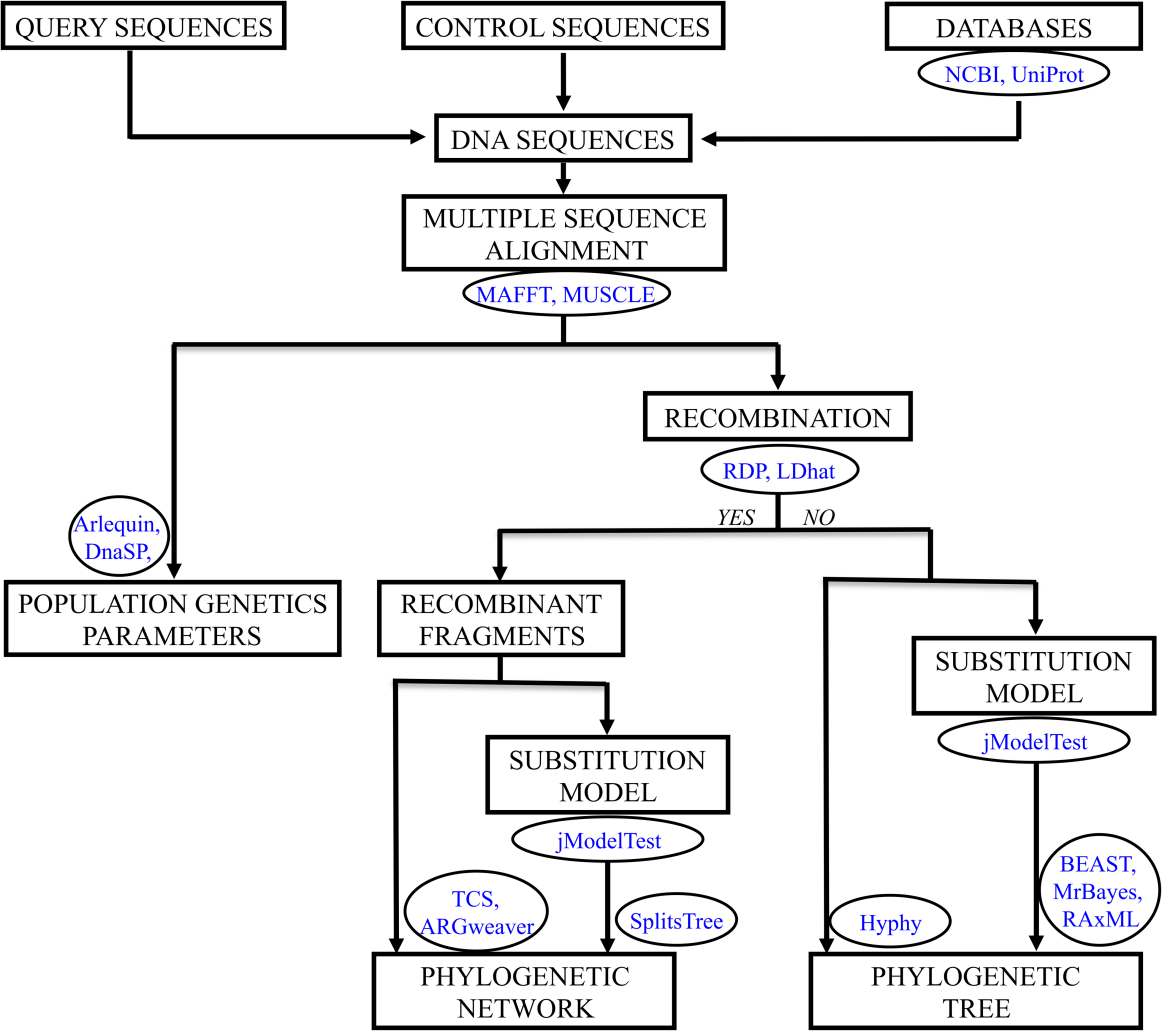
Pipeline showing the evolutionary analysis of genetic data oriented to NHFG. Data and tasks are shown in boxes, and databases and computer frameworks are shown in circles. Population genetic parameters include measures of genetic diversity, genetic differentiation, and demographics. The phylogenetic analysis requires the previous identification of recombination and can be performed ignoring or considering a substitution model of evolution. NHFG, nonhuman forensic genetics.

The computational pipeline for the evolutionary analysis of genetic data in NHFG follows well-established methodologies (Fig 3); however, several steps must be carefully performed. First, query, control (from local and background regions), and external (i.e., from reliable databases) sequences must be aligned. Next, population genetics statistics such as genetic diversity and genetic differentiation (i.e., between query and control sequences) can be estimated [e.g., 54, 55, 56]. The alignment can also be used to infer a phylogenetic history that depicts genetic relationships between the sample sequences and provides the timing of common ancestors (i.e., transmission events). A large number of pathogens (including those involved in most of NHFG cases, Human Immunodeficiency Virus [HIV] and Hepatitis C Virus [HCV]) evolve with processes of exchange of genetic material such as recombination [57, 58] and horizontal gene transfer [59]. Importantly, ignoring these processes can bias phylogenetic tree inferences by generating incorrect branch lengths and topologies [60, 61]. Therefore, under the presence of these processes, a phylogenetic network, which may have embedded a phylogenetic tree for each exchanged fragment [62], should be inferred [35, 61, 63, 64]. Indeed, a substitution model of evolution that fits the data best should be selected and considered in sophisticated phylogenetic inferences (i.e., based on maximum-likelihood or Bayesian approaches) [61, 65]. Importantly, phylogenetic approaches usually implement statistical confidence of the inferred evolutionary relationships through a bootstrap analysis [66]. In NHFG, this statistical parameter can provide a measure of the reliability of relationships between the pathogen genetic sequences of the investigated individuals. For example, a number of forensic studies based on phylogenetic inferences showed a classification of all control individuals in significantly separated clades, whereas individuals related with the studied outbreak or transmission clustered in a unique clade [e.g., 31, 32, 34, 35, 53, 54, 55]. Likelihood ratio tests can also be useful for hypothesis testing (i.e., testing if control sequences group or not with the studied outbreak) [e.g., 54].

As noted above, the estimated time of internal nodes of the inferred phylogeny can be useful in forensic litigations by revealing the timing of infections [52]. These times can be estimated with Bayesian approaches [e.g., 67] accounting for longitudinal sampling (the tips are dated with the corresponding sampling times) to calibrate the (often relaxed) molecular clock and can provide accurate confidence intervals [e.g., 54].

## Applications of NHFG

NHFG is expanding to more and more biological areas due to the increasing emergence of forensic cases based on NHGM. In this section, we revise the most relevant areas of NHFG, including zoology, botany, microbiology, and food analysis and traceability.

## Zoology

The relevance and close presence of animals in a variety of human activities explain why they are among the first targets of NHFG [6, 68–70]. The number of animal species studied from a forensic genetics perspective has increased significantly, and different testing protocols have been developed for determining the identity of a sample at different biological levels such as individual, population, breed, species, or higher taxonomic classifications.

The preferential DNA markers used for individual identification in animals are autosomal STRs, as established in HFG. For example, STR kits have been developed for individual identification and kinship testing in dogs [71–78], cats [79–81], horses [40, 82, 83], cattle [39, 84], bears [85], deer [86, 87], badgers [88], birds [89, 90], and koi carps [91]. They have also been employed in resolving criminal and civil cases, such as dog or bear attacks [37, 38, 92], silent witnesses of crimes [6], identification of samples from sport horses [41, 93], and in wildlife crime investigations (wildlife forensics), including big cats [94], mouflons [95, 96], wild boars [97, 98], and elephants [99], among others. Concerning the latter, we want to highlight the application of forensic genetics to the illegal wildlife trade (IWT), since this is one of the biggest threats to a variety of species and habitats, with a consequent loss of biodiversity [100, 101]. In addition, IWT is a large-scale business estimated in billions of euros that generate negative socioeconomic impacts [100, 101]. Importantly, forensic genetics plays a crucial role in wildlife law enforcement [101].

Pioneer works endured tremendous efforts trying to reach the quality standards of human genetic testing. Difficulties in developing a new genotyping system for animals are various, including the collection of representative samples (especially problematic in wild species), the access to high-quality genomic sequences (not available for several species) and obtaining funding for the experiments (often focused on human research). Therefore, some of these STR kits are still a few steps behind those developed for human identification. For example, dinucleotide repeats are still used in nonhuman DNA testing [e.g., 37, 88, 91, 94], making it difficult to interpret sample mixtures and heterozygotes due to stutter product formation [102]. The most advanced nonhuman profiling kits are those developed for domesticated animals, including several STRs with tetranucleotide repeats [e.g., 72, 80, 81, 103]. Indeed, sex chromosome STR markers can also be useful for NHFG, however they still remain uncharacterized for many animal species. The mammalian Y-chromosome is used for gender identification, resolving paternity and family structures with application in forensic investigations [e.g., 104, 105, 106]. The development of an X-chromosome STR kit for dogs in 2010 [107] was a promising step in this field but, unfortunately, it was not followed by similar works in other species. The determination of the sex in birds has been possible using markers located in the W and Z chromosomes [e.g., 108, 109, 110].

A few panels of autosomal SNPs have also been developed for individual identification in different animal species [111–117]. These genetic markers may have some technical advantages over STRs [e.g., 102, 118] and can provide information about physical traits.

Forensic zoology often has to deal with degraded samples. In such cases, mtDNA may be the only source of genetic information that can be used. The high copy number of mtDNA in cells increases the probability of obtaining results from degraded/low-copy DNA samples such as hair, bones, and scat [119, 120]. Importantly, the same mtDNA sequence can be found in many individuals of a population and therefore cannot be used for individual identification. However, it can be used to exclude an individual as a source of a casework sample, and its utility has been demonstrated for a variety of animal species [e.g., 70, 121, 122–124]. Nevertheless, the most successful use of mtDNA in forensic zoology has been in species identification. Different mtDNA regions have been tested and validated for use in a forensic context, including CYTB [125–127], COI [128–130], and rRNA genes [131, 132]. The procedure usually involves the sequencing of a variable region amplified with conserved PCR primers followed by database searches and phylogenetic analyses. This strategy was applied in different forensic investigations such as identification of rhinoceros horns [133], ivory [134], turtle shells [135], endangered snake species [136], tigers [137], forensically important insect species [138–140], illegally smuggled eggs [141], or fish and fish products [142–144]. A few multiplex PCR/primer extension assays to genotype mtDNA SNPs have also been developed for species and subspecies identification (i.e., tiger [49], elephant [145], and other animals [146, 147]).

While the genetic identification of an individual or a species is not problematic in most situations, defining animal breeds or geographical populations has been considerably more difficult. Most breeds had a recent origin and are often defined by a few morphological features arbitrarily defined. For instance, cat breeds are defined by phenotypic characteristics (e.g., hair length, coat patterning, and colours) that are single-gene traits. Nevertheless, most cats can be assigned to their proper breed or population of origin using genetic data [148].

A crucial aspect for some forensic cases (i.e., poaching or illegal logging) is the identification of the origin of the sample. This identification depends on the existence of genetic data in different regions (including the region of the “real” origin), enough genetic differentiation among regions and the quality of the analytical method. The recent origin, intensive inbreeding, and genetic drift make difficult-to-use neutral genetic markers for rigorous identification of breed or populations. In such ambiguous cases, genetic tests should assess the genetic variants of the morphological traits that define the breed. However, our understanding of the genetics underlying such complex traits is still very limited, although significant progress is expected [149]. A famous case of origin identification was the mad cow disease between the United States and Canada [details in 150, 151], where a novel parentage testing was developed by combining prions and kinship [151].

## Botany

Plant evidence can provide crucial information for the reconstruction of forensically relevant events or in cases where the crime scene and autopsy reports are not compelling [152].

Conventional taxonomic identification (using morphological methods) has a reduced application since botanical forensic evidences are often very fragmented (e.g., pieces of leaves or seeds) limiting the use of dichotomous keys. However, molecular markers can be applied to identify samples, regardless of their state, morphology, and development phase. In this concern, in the last couple of decades, diverse molecular markers have been applied for the forensic identification of species and individuals (i.e., HRM coupled with specific barcodes or real-time PCR to analyze chloroplast DNA regions) [e.g., 153, 154]. Importantly, second- and third-generation sequencing methodologies are providing affordable analysis of complex and degraded plant samples [155, 156].

Forensic botany presents numerous applications such as the identification of the origin of seized illegal drugs (marijuana [157], kratom [158], or opium [159]), detection of illegal logging [160, 161], importation and commercialization of endangered and exotic species [162, 163], or bioterrorism (abrin and ricin attacks [164]). It can also provide useful supporting evidence in crime scene investigations, allowing us to establish a link between the victim and the suspect, placing the suspect at a crime scene or estimating the time of death [e.g., 23, 25, 165–167].

## Microbiology

Although microbes have long been recognized as important players in our daily life, present in areas such as medicine and public health, ecology, and in industrial applications, microbial forensics (MF) is still a relatively recent scientific field [168, 169].

MF aims to identify a target microorganism and its source. Although culture in selective growth media remains as the preferred standard for characterization of microbial agents at the resolution of genus/species level, complementary detection methods based on diverse molecular markers are increasingly applied [170]. Indeed, NGS technologies profoundly improved the ability to detect microorganisms, even when present in low abundance or in degraded or mixture samples, and to differentiate at strain/isolate level, using diagnostic genomic signatures [171].

Applications of MF involve diverse areas such as biocrimes, bioterrorism, frauds, outbreaks and transmission of pathogens, or accidental release of a biological agent or a toxin [e.g., 54, 172]. Additionally, the recent breakthroughs derived from NGS technologies allowed the analysis of microbial evidence to be expanded to cases related with geolocation, body fluid characterization, or postmortem interval estimation [168].

Some biological agents can be used as weapons or threats. The best well-known example is the Amerithrax case (2001), where letters laden with *Bacillus anthracis* spores were sent through the U.S. Postal Service to several media offices in New York and Florida and to U.S. senators in Washington [173, 174]. In this case, DNA evidence was found in the suspect’s laboratory.

Under the scope of epidemiological investigation, MF also helps to determine whether a pathogen outbreak was natural or human-driven. Therefore, MF is intimately associated with epidemiological surveys, allowing studying and following disease outbreak dynamics, mainly concerning the identification of the agent or toxin, origin and natural reservoirs, genetic diversity and evolution, and possible transmission routes. Some well-known cases of the epidemiological studies are the swine-origin influenza A virus (H1N1; 2009) [175], the Haitian cholera (2010) [176], the haemolytic-uremic syndrome (*Escherichia coli* O104:H4; 2011) [177], the Coronavirus Middle East respiratory syndrome (2012) [178], the avian-origin Influenza A virus (H7N9; 2013) [179], the West African Ebola virus (2013/2015) [180], the Middle Eastern poliomyelitis (2014) [181], the Portuguese Legionnaires’ disease (2014) [182], and the Zika virus outbreaks [183]. Note that most of the cases indicated above applied NGS approaches to identify and study the different biological agents.

Applications of MF in biocrimes also include the tracking of sexually transmitted diseases and healthcare malpractice linked to the transmission of HIV [e.g., 31–36, 53, 184] and HCV [e.g., 54, 55]. Moreover, this discipline is also used to determine responsibilities in cases of hospital-acquired infections [e.g., 185, 186] or sudden death syndrome [e.g., 187, 188].

The human microbiome is starting to be a focus of interest for identification purposes. The rational is to trace human microbiomes on our skin on the surfaces and objects we interact with the potential to supplement the use of human DNA for associating people with evidence and environments. The Human Microbiome Project has significantly improved the scientific knowledge in the field [189, 190]. Note that there are 10 times more bacteria than human cells in our body [191], and a number of them appears to be unique to each person [192], offering an opportunity for new identification biomarkers [193]. Thus, the human microbiome could be used to identify suspects [e.g., 27, 28, 29] and to estimate the postmortem interval [194]. For example, the origin of human remains from the Second World War was ascertained with the parvovirus B19V [195]. Although these are promising findings, we consider that we are still far from a foundational validation of this approach to be used in legal cases.

One of the main constraints associated with the use of MF is the lack of standards and guidelines, although phylogenetic analyses have supported associations and have successfully been admitted as evidence in legal criminal cases [196]. Another limitation is the insufficiency of reference databases lacking endemic data or microorganism source tracing, reference genome sequences, metadata, and representative genetic diversity coverage [197].

## Food analysis and traceability

The investigation of the biological composition of food products regarding the species, variety or cultivar, and geographic origin is of major forensic interest. Such investigations are relevant for guaranteeing consumer choices according to health concerns (e.g., sensitivities or allergies), dietary preferences (e.g., vegetarian, nongenetically modified organisms), religious beliefs (e.g., halal and kosher specifications), and to detect fraudulent substitution of a given species by a similar one with lower economic value [198, 199]. Labelling is indispensable for producers, retailers, and consumers to recognize and validate components of foodstuffs [200]. Unfortunately, labels of products often provide insufficient and erroneous information concerning the exact contents.

The methodology used in food forensics is similar to that used in classical crime investigations, facing the same demands of dealing with potentially degraded DNA samples [201]. Several DNA-based methods have become remarkably valuable for protecting and certifying the quality and source of food [202, 203]. The first studies performed in the 90’s resorted to classical techniques (i.e., RADP and ISSR) but nowadays, real-time PCR [204], HRM [205–207], and MPS [208] are widely applied for food traceability with the advantage of quantifying each particular component in a faster and affordable procedure.

These genetic markers have been applied to perform identification in a variety of food products such as olive oil [e.g., 209, 210], grapevine cultivars [e.g., 211, 212, 213], composition of honey [e.g., 214, 215, 216], mushrooms [e.g., 217, 218, 219], dairy products [e.g., 220, 221, 222], seafood products [20], or meat species adulteration [223]. Additional documented cases include: *i*) identification of cultivars of basmati rice [224], pome [225] and stone fruits [226], leguminosae [227, 228], coffee [229], and tea and infusions [230]; *ii*) patent misappropriation of strawberry cultivars [231]; *iii*) confirmation of Protected Designation of Origin (PDO), Protected Geographical Indication (PGI), or Traditional Speciality Guaranteed (TSG) in olive [232] and grape [213, 233] products; *iv*) adulteration of traditional medicines [234, 235] and herbs or spices [236]; *v*) insufficient and erroneous food labelling, including the presence of some hidden allergens [237, 238] or genetically modified organisms [239] (GMOs; see section Genetically modified organisms).

### Food microbiology

Over the last 2 decades, the prevalence of foodborne diseases has drastically increased, becoming a worldwide major public health concern. Foodborne diseases are often triggered by the consumption of food or water contaminated either by pathogens (bacteria, viruses, fungi, and parasites) or derived toxins. The most common pathogens responsible for foodborne disease outbreaks are *Listeria monocytogenes*, *Escherichia coli* O157:H7, *Staphylococcus aureus*, *Salmonella enterica*, *Bacillus cereus*, *Vibrio* spp., *Campylobacter jejuni*, *Clostridium perfringens*, and *Shigella dysenteriae*. These pathogens are often associated with consumption of raw (e.g., fruits and vegetables) or undercooked foods (e.g., seafood, meat, and poultry) [240]. To overcome the limitations of the traditional culture of microorganisms (e.g., may disallow the cultivation of the major foodborne pathogen or may present a slow growth leading to long periods of time cultivation), DNA/RNA-based methods (i.e., STR, NASBA, LAMP, and NGS) are usually applied [e.g., 241].

### Genetically modified organisms

An area of growing interest is the detection of GMOs. The number of genetically modified plants has been growing in recent years despite the intense discussion about the benefits or damage that these organisms may have on humans and ecosystems. The detection of a GMO is carried out by targeting the genetic elements (promotors, protein-coding regions or terminators) that have been introduced artificially in the genome of the transgenic organism in order to improve a particular trait [242]. A curated list of transgenic reference sequences has been recently made available and is expected to facilitate the development of methods for testing GMOs and the implementation of regulatory policies [243]. The labelling and traceability of GMOs are important issues that are highly regulated. If the content exceeds a certain threshold, the product must be labeled accordingly. The most commonly used DNA-based methodology for GMO testing is PCR, although other techniques have been proposed [244–246]. The quantification of DNA targets is usually done by real-time PCR, where the copy number of the transgenic element detected is correlated to a common plant marker, allowing the determination of the GMO proportion in the sample [247, 248]. The correct detection of genetically modified materials is of forensic relevance not only due to strict legislation regarding the labelling of food products but also due to the type of materials from which DNA has to be extracted. For example, transgenic constructs have to be identified in DNA extracted from products like corn germ, flour, pasta, corn flakes, cookies, baked products, sugars derived from corn starch, soy cream or milk (liquid or lyophilized), tofu, meat products, lecithin, and even oil. Although most of the currently available GMOs are plants, the picture is expected to change soon. The first genetically modified animal (AquAdvantage salmon) is on the verge of being approved for human consumption in different countries [249]. New methods are being developed to detect the genetically modified salmon in food products [250, 251]. Strong legislation is expected to regulate the presence of this transgenic animal in foods and environmental samples [252, 253] and, consequently, reliable and sensitive methods for its detection will be required by regulatory and scientific agencies worldwide [245, 254].

## The future of NHFG

Within the enormous variety of applications, methods, and sources of NHFG, the forensic use of NHGM is still limited and faces enormous difficulties due to diverse causes. Among them, and perhaps the most important, is the sheer amount of biodiversity and the current poor knowledge about it, with an impact not only on the species identification problem but also at the intraspecific level where, for most wildlife organisms, population genetics data are nonexistent or extremely poor. This makes relevant parameters difficult to estimate with acceptable accuracy and thus inhibits solid statistical evaluation of the evidence [255]. In this concern, the impact of the International Barcode of Life project (iBOL, http://www.ibol.org/) on forensics has been much less than desired and several difficulties have been raised on its power, limitations, and governance [256, 257]. In fact, most biodiversity studies do not meet classical forensic standards (demanded in forensic routine casework), due to the inherently limited sampling, references and controls. Moreover, there is a lack of agreement and concerted actions between the scientific societies aiming at the forensic use of NHGM (ISAG, International Society for Animal Genetics; ISFG, International Society for Forensic Genetics; SWSF, The Society for Wildlife Forensic Science; ISEF, International Society of Environmental Forensics) that is reflected in nonreconcilable or even contradictory recommendations and guidelines (particularly between ISFG [9] and ISAG/FAO [258]). Given the increasing use of NHFG, we do hope for some progress in joining efforts between scientific communities for a mutual benefit.

On the other hand, less error-burdened, cheaper, and faster MPS, together with progress in bioinformatics frameworks and computational resources, now allow the analysis of complex samples (i.e., commingled samples with DNA from more than one contributor/species) with more accurate and reliable results [e.g., 177, 186]. With third generation sequencing technologies, single DNA molecules can be analyzed individually [e.g., 259] and, therefore, haplotypes can be determined. These advances are expected to revolutionize NHFG. Among other examples, MPS was already applied to the identification of species for quality control in the development and authentication of herbal and traditional medicines [260] and for the discrimination of soils and other detritus from alternative environments and locations, based on the composition of the microflora, plants, metazoan, and protozoa DNA sequences [21, 261–265]. As noted in MF, the implementation of MPS is particularly useful for epidemiological studies. However, more research is necessary for the improvement of libraries (i.e., reference sequences reflecting the coverage of the entire genome of diverse organisms), development of bioinformatics platforms (i.e., for decreasing memory requirements and implementing algorithms for parallel computing) and for reproducibility and assignment of general quality of the results. Moreover, current MPS technologies still present relatively high sequencing errors [e.g., 266] which, although could be assumed for other disciplines, may not meet forensic standards [267]. Therefore, strict MPS validation studies are mandatory but they are still very scarce, even in human applications.

In this regard, it is clear that genetic analyses based on very large datasets (ideally, whole genomes) can provide high statistical confidence that can be useful for forensic cases [268]. However, systematic biases in methods applied to the analysis of large data can lead to precise but incorrect results [269–271]. Therefore, not only are large datasets required, less biased state-of-the-art methodologies should also be applied [272]. An example of this situation is the evolutionary analysis of genetic data. This analysis can be improved with the consideration of more complex substitution models of evolution (i.e., nonreversible and nonstationary) that can better fit the data [65, 273]. However, these models were not implemented yet into the traditional frameworks of the phylogenetic pipeline and, to our knowledge, all existing NHFG studies have ignored them. In addition, as noted above, evolutionary processes that exchange genetic material (e.g., recombination) can bias phylogenetic tree inferences [60]. However, to our knowledge, all existing NHFG studies including phylogenetic tree inferences from pathogens that usually evolve with high recombination rates (i.e., HIV and HCV) ignored recombination [e.g., 31–36, 53, 54, 55]. We strongly recommend considering these aspects in future NHFG studies.

The future of NHFG is dependent on the progress in removing current limitations (i.e., funding, adapting scientific methods into court [274], taking away from HFG and dealing with much smaller documented biodiversity being more complex to achieve forensic standards), but this is an emerging field of increasing importance. The number of papers in the top forensic journals on nonhuman DNA typing topics is increasing at a rate of 15% per year, especially on IWT [275]. As mentioned by Ogden and Linacre [101], perhaps the main difficulty in this field is the large proportion of traded products originated from underdeveloped countries where wildlife trade monitoring and the ability of the enforcement agencies to act are limited. That difficulty is caused by the lack of funding since the priorities of the majority of law enforcement agencies are crimes against humans and their properties.

The continuous incorporation of genomic data in reliable databases together with progress of experimental methodologies and analytical software are expected to further increase the application of NHFG. Assuming this direction, we believe that, in the future, NHFG could even overpass HFG in number of cases investigated, since the number of informative organisms is extremely large.

## Supporting information

S1 Table Illustrative examples of scenarios using nonhuman genetic material (NHGM) for auxiliary evidence in litigations related with human identification. (PDF)

S2 Table Illustrative examples of scenarios of nonhuman genetic material (NHGM) for main evidence in litigations not related with human identification. (PDF)

S1 Text Literature cited in the supplementary material. (PDF)
