# Supplementary material for: Forensic genetics and genomics: Much more than just a human affair
Source: PLoS Genet. 2017 Sep 21;13(9):e1006960. doi: 10.1371/journal.pgen.1006960 (PMC5608170; doi:10.1371/journal.pgen.1006960)
Supplement: S1 Table — (PDF) [file pgen.1006960.s001.pdf]

Application	Type of source	Organism or material (additional information)	Reference
Associative evidence in criminalistics	Animals	Cat (implication of murder suspect)	[1, 2]
Associative evidence in criminalistics	Plants	Oak (evidence in criminal case)	[3]
		Knotgrass (evidence in criminal case)	[4]
		Mosses (evidence in criminal case)	[5]
		Soil and plants (selection between alternative paths)	[6]
		Amanita phalloides and yew (poisoning)	[7, 8]
Post-mortem interval		Bryophytes and plant roots (identification of post-mortem time interval)	[9]
Post-mortem interval	Insects	Calliphoridae (identification of post-mortem time interval)	[10]
Associative evidence in criminalistics	Bacteria	Skin bacterial communities (identification of individual)	[11, 12]
		Salivary bacterial communities (identification of individual)	[13]
		Gut bacterial communities (identification of individual)	[14]
		Staphylococcus aureus and *Clostridium difficile* (outbreak detection)	[15, 16]
		Bacillus anthracis (bioterrorism)	[17-19]
		Profiles of footwear insoles (forensic discrimination)	[20]
Associative evidence in criminalistics	Virus	Hepatitis C (identification of virus outbreak)	[21, 22]
		HIV-1 (source identification in criminal cases)	[23, 24]
		HIV-1 (deliberated transmission)	[25-33]

		HIV-1 (lack of transmission from the suspect)	[34]
Associative evidence in criminalistics	Admixture	Soil DNA (evidence in murder case)	[35]
