# Supplementary material for: Forensic genetics and genomics: Much more than just a human affair
Source: PLoS Genet. 2017 Sep 21;13(9):e1006960. doi: 10.1371/journal.pgen.1006960 (PMC5608170; doi:10.1371/journal.pgen.1006960)
Supplement: S2 Table — (PDF) [file pgen.1006960.s002.pdf]

Type of case	Application	Organism or material (additional information)	Reference
Individualization/ kinship	Death/bodily/materia l harm	Bear (attack)	[36]
		Dog (attack)	[37, 38]
		Dog (traffic accident)	[39]
		Bird (aircraft collision)	[40]
	Identification	Horse (race doping control)	[41]
	Kinship/Pedigree certification	Cattle (paternity)	[42]
		Horse (paternity)	[43]
		Dog (paternity and pedigree)	[44-46]
		Pigeon (paternity)	[47]
Taxonomic/speci es assignment	Wildlife/protected species	Smuggled eggs (identification)	[48]
		Big cats (identification)	[49]
		Deer (identification)	[50]
		Tiger (poaching)	[51]
		Mouflon (poaching)	[52, 53]
		Bear (poaching)	[54]
		Coniferous trees (illegal logging)	[55]
		Palm (illegal trading)	[56]
	Identification of totally unknown species	Stomach contents (suicidal poisoning)	[8]
		Unknown animal (bite through a tent)	[57]
		Unknown livestock predators	[58, 59]
	Food and drug analyses	Dairy products (authentication)	[60-63]
		Caviar (mislabeling)	[64, 65]
		Meat products (detection	[66, 67]

		of species origin)	
		Meat (detection of religiously forbidden species)	[68]
		Fish and seafood products (detection of species origin)	[69, 70]
		Olive oils (authentication)	[71-73]
		Honey (authentication)	[74]
		Herbal teas (authentication)	[75, 76]
		Coffee (authentication)	[77]
		Drugs (legality and safety of traditional medicines)	[78, 79]
		Food traceability (food safety and identification of commercial fraud)	[80-85]
	Drug enforcement	Cannabis (identification)	[86, 87]
Population/breed/ strain/variety assignment	Patent violation	Strawberry varieties (identification)	[88]
	Livestock robbery	Cattle rustling (identification)	[89]
		Cattle (illegal purchase of stolen animals)	[90]
	Fishing, competition and fraud	Salmon populations	[91]
		Commercial marine fish (false eco-certification)	[92]
		Koi carp (illegal fishing)	[93]
	Ivory source	Ivory (identification)	[94-96]
	Breeds identification	Horses	[43]
		Sheep	[97]
		Beef cattle	[98]
		Pigs	[99]
		Dogs	[100]
	Authenticity	Grapevine varieties	[101]

		Wine	[102]
Geographic assignment	Dust analysis	Fungi	[103]
	Identification of geographic variants	Blow fly	[104]
	Identification of geographic variants. Global biosecurity	Insects	[105]
Detection of genetically modified organisms (GMO)	GMO identification	Food products	[106]
