# Supplementary material for: Forensic genetics and genomics: Much more than just a human affair
Source: PLoS Genet. 2017 Sep 21;13(9):e1006960. doi: 10.1371/journal.pgen.1006960 (PMC5608170; doi:10.1371/journal.pgen.1006960)
Supplement: S1 Text — (PDF) [file pgen.1006960.s003.pdf]

1. Menotti-Raymond MA, David VA, O'Brien SJ. Pet cat hair implicates murder suspect. Nature. 1997;386(6627):774. doi: 10.1038/386774a0. PubMed PMID: 9126735.
2. Branicki W, Kupiec T, Pawlowski R. Validation of cytochrome b sequence analysis as a method of species identification. J Forensic Sci. 2003;48(1):83-7. PubMed PMID: 12570204.
3. Craft KJ, Owens JD, Ashley MV. Application of plant DNA markers in forensic botany: genetic comparison of Quercus evidence leaves to crime scene trees using microsatellites. Forensic Sci Int. 2007;165(1):64-70. doi: 10.1016/j.forsciint.2006.03.002. PubMed PMID: 16632287.
4. Koopman WJ, Kuiper I, Klein-Geltink DJ, Sabatino GJ, Smulders MJ. Botanical DNA evidence in criminal cases: Knotgrass (Polygonum aviculare L.) as a model species. Forensic Sci Int Genet. 2012;6(3):366-74. doi: 10.1016/j.fsigen.2011.07.013. PubMed PMID: 21880564.
5. Korpelainen H, Virtanen V. DNA fingerprinting of mosses. J Forensic Sci. 2003;48(4):804-7. PubMed PMID: 12877296.
6. Aquila I, Ausania F, Di Nunzio C, Serra A, Boca S, Capelli A, et al. The role of forensic botany in crime scene investigation: case report and review of literature. J Forensic Sci. 2014;59(3):820-4. doi: 10.1111/1556-4029.12401. PubMed PMID: 24502402.
7. Gausterer C, Penker M, Krisai-Greilhuber I, Stein C, Stimpfl T. Rapid genetic detection of ingested Amanita phalloides. Forensic Sci Int Genet. 2014;9:66-71. doi: 10.1016/j.fsigen.2013.11.002. PubMed PMID: 24528582.
8. Gausterer C, Stein C, Stimpfl T. Application of direct PCR in a forensic case of yew poisoning. Int J Legal Med. 2012;126(2):315-9. doi: 10.1007/s00414-011-0607-0. PubMed PMID: 21805302.
9. Cardoso HF, Santos A, Dias R, Garcia C, Pinto M, Sergio C, et al. Establishing a minimum postmortem interval of human remains in an advanced state of skeletonization using the growth rate of bryophytes and plant roots. Int J Legal Med. 2010;124(5):451-6. doi: 10.1007/s00414-009-0372-5. PubMed PMID: 19714355.
10. Tuccia F, Giordani G, Vanin S. A general review of the most common COI primers for Calliphoridae identification in forensic entomology. Forensic Sci Int Genet. 2016;24:e9-e11. doi: 10.1016/j.fsigen.2016.07.003. PubMed PMID: 27444889.
11. Fierer N, Lauber CL, Zhou N, McDonald D, Costello EK, Knight R. Forensic identification using skin bacterial communities. Proc Natl Acad Sci U S A. 2010;107(14):6477-81. doi: 10.1073/pnas.1000162107. PubMed PMID: 20231444; PubMed Central PMCID: PMCPMC2852011.
12. Yao X, Liu W, Han J, Pei G, Tong Y, Luo Y. Analysis of Microbiome DNA on Frequently Touched Items and from Palm Prints. Journal of Forensic Science and Medicine. 2016;2(2):74-7. doi: 10.4103/2349-5014.184190.
13. Leake SL, Pagni M, Falquet L, Taroni F, Greub G. The salivary microbiome for differentiating individuals: proof of principle. Microbes Infect. 2016;18(6):399-405. doi: 10.1016/j.micinf.2016.03.011. PubMed PMID: 27063111.
14. Xie H, Guo R, Zhong H, Feng Q, Lan Z, Qin B, et al. Shotgun Metagenomics of 250 Adult Twins Reveals Genetic and Environmental Impacts on the Gut Microbiome. Cell Syst. 2016;3(6):572-84 e3. doi: 10.1016/j.cels.2016.10.004. PubMed PMID: 27818083.

15. Eyre DW, Golubchik T, Gordon NC, Bowden R, Piazza P, Batty EM, et al. A pilot study of rapid benchtop sequencing of Staphylococcus aureus and Clostridium difficile for outbreak detection and surveillance. BMJ Open. 2012;2(3). doi: 10.1136/bmjopen-2012-001124. PubMed PMID: 22674929; PubMed Central PMCID: PMCPMC3378946.
16. Koser CU, Holden MT, Ellington MJ, Cartwright EJ, Brown NM, Ogilvy-Stuart AL, et al. Rapid whole-genome sequencing for investigation of a neonatal MRSA outbreak. N Engl J Med. 2012;366(24):2267-75. doi: 10.1056/NEJMoa1109910. PubMed PMID: 22693998; PubMed Central PMCID: PMCPMC3715836.
17. Rasko DA, Worsham PL, Abshire TG, Stanley ST, Bannan JD, Wilson MR, et al. Bacillus anthracis comparative genome analysis in support of the Amerithrax investigation. Proc Natl Acad Sci U S A. 2011;108(12):5027-32. doi: 10.1073/pnas.1016657108. PubMed PMID: 21383169; PubMed Central PMCID: PMCPMC3064363.
18. Jernigan JA, Stephens DS, Ashford DA, Omenaca C, Topiel MS, Galbraith M, et al. Bioterrorism-related inhalational anthrax: the first 10 cases reported in the United States. Emerg Infect Dis. 2001;7(6):933-44. doi: 10.3201/eid0706.010604. PubMed PMID: 11747719; PubMed Central PMCID: PMCPMC2631903.
19. Takahashi H, Keim P, Kaufmann AF, Keys C, Smith KL, Taniguchi K, et al. Bacillus anthracis incident, Kameido, Tokyo, 1993. Emerg Infect Dis. 2004;10(1):117-20. doi: 10.3201/eid1001.030238. PubMed PMID: 15112666; PubMed Central PMCID: PMCPMC3322761.
20. Goga H. Comparison of bacterial DNA profiles of footwear insoles and soles of feet for the forensic discrimination of footwear owners. Int J Legal Med. 2012;126(5):815-23. doi: 10.1007/s00414-012-0733-3. PubMed PMID: 22729347.
21. Gonzalez-Candelas F, Bracho MA, Wrobel B, Moya A. Molecular evolution in court: analysis of a large hepatitis C virus outbreak from an evolving source. BMC Biol. 2013;11:76. doi: 10.1186/1741-7007-11-76. PubMed PMID: 23870105; PubMed Central PMCID: PMCPMC3717074.
22. Gonzalez-Candelas F, Bracho MA, Moya A. Molecular epidemiology and forensic genetics: application to a hepatitis C virus transmission event at a hemodialysis unit. J Infect Dis. 2003;187(3):352-8. doi: 10.1086/367965. PubMed PMID: 12552418.
23. Scaduto DI, Brown JM, Haaland WC, Zwickl DJ, Hillis DM, Metzker ML. Source identification in two criminal cases using phylogenetic analysis of HIV-1 DNA sequences. Proc Natl Acad Sci U S A. 2010;107(50):21242-7. doi: 10.1073/pnas.1015673107. PubMed PMID: 21078965; PubMed Central PMCID: PMCPMC3003064.
24. Machuca R, Jorgensen LB, Theilade P, Nielsen C. Molecular investigation of transmission of human immunodeficiency virus type 1 in a criminal case. Clin Diagn Lab Immunol. 2001;8(5):884-90. doi: 10.1128/CDLI.8.5.884-890.2001. PubMed PMID: 11527797; PubMed Central PMCID: PMCPMC96165.
25. Ou CY, Ciesielski CA, Myers G, Bandea CI, Luo CC, Korber BT, et al. Molecular epidemiology of HIV transmission in a dental practice. Science. 1992;256(5060):1165-71. PubMed PMID: 1589796.
26. Siljic M, Salemovic D, Cirkovic V, Pesic-Pavlovic I, Ranin J, Todorovic M, et al. Forensic application of phylogenetic analyses - Exploration of suspected HIV-1 transmission case. Forensic Sci Int Genet. 2017;27:100-5. doi: 10.1016/j.fsigen.2016.12.006. PubMed PMID: 28024238.

27. Albert J, Wahlberg J, Leitner T, Escanilla D, Uhlen M. Analysis of a rape case by direct sequencing of the human immunodeficiency virus type 1 pol and gag genes. J Virol. 1994;68(9):5918-24. PubMed PMID: 7520096; PubMed Central PMCID: PMCPMC236997.
28. Veenstra J, Schuurman R, Cornelissen M, van't Wout AB, Boucher CA, Schuitemaker H, et al. Transmission of zidovudine-resistant human immunodeficiency virus type 1 variants following deliberate injection of blood from a patient with AIDS: characteristics and natural history of the virus. Clin Infect Dis. 1995;21(3):556-60. PubMed PMID: 8527543.
29. Metzker ML, Mindell DP, Liu XM, Ptak RG, Gibbs RA, Hillis DM. Molecular evidence of HIV-1 transmission in a criminal case. Proc Natl Acad Sci U S A. 2002;99(22):14292-7. doi: 10.1073/pnas.222522599. PubMed PMID: 12388776; PubMed Central PMCID: PMCPMC137877.
30. Lemey P, Van Dooren S, Van Laethem K, Schrooten Y, Derdelinckx I, Goubau P, et al. Molecular testing of multiple HIV-1 transmissions in a criminal case. AIDS. 2005;19(15):1649-58. PubMed PMID: 16184035.
31. Birch CJ, McCaw RF, Bulach DM, Revill PA, Carter JT, Tomnay J, et al. Molecular analysis of human immunodeficiency virus strains associated with a case of criminal transmission of the virus. J Infect Dis. 2000;182(3):941-4. doi: 10.1086/315751. PubMed PMID: 10950794.
32. Chen M, Ma Y, Yang C, Yang L, Chen H, Dong L, et al. The combination of phylogenetic analysis with epidemiological and serological data to track HIV-1 transmission in a sexual transmission case. PLoS One. 2015;10(3):e0119989. doi: 10.1371/journal.pone.0119989. PubMed PMID: 25807147; PubMed Central PMCID: PMCPMC4373787.
33. Goujon CP, Schneider VM, Grofti J, Montigny J, Jeantils V, Astagneau P, et al. Phylogenetic analyses indicate an atypical nurse-to-patient transmission of human immunodeficiency virus type 1. J Virol. 2000;74(6):2525-32. PubMed PMID: 10684266; PubMed Central PMCID: PMCPMC111740.
34. Jaffe HW, McCurdy JM, Kalish ML, Liberti T, Metellus G, Bowman BH, et al. Lack of HIV transmission in the practice of a dentist with AIDS. Ann Intern Med. 1994;121(11):855-9. PubMed PMID: 7978698.
35. Concheri G, Bertoldi D, Polone E, Otto S, Larcher R, Squartini A. Chemical elemental distribution and soil DNA fingerprints provide the critical evidence in murder case investigation. PLoS One. 2011;6(6):e20222. doi: 10.1371/journal.pone.0020222. PubMed PMID: 21674041; PubMed Central PMCID: PMCPMC3108598.
36. Frosch C, Dutsov A, Georgiev G, Nowak C. Case report of a fatal bear attack documented by forensic wildlife genetics. Forensic Sci Int Genet. 2011;5(4):342-4. doi: 10.1016/j.fsigen.2011.01.009. PubMed PMID: 21315676.
37. Tsuji A, Ishiko A, Kimura H, Nurimoto M, Kudo K, Ikeda N. Unusual death of a baby: a dog attack and confirmation using human and canine STRs. Int J Legal Med. 2008;122(1):59-62. doi: 10.1007/s00414-006-0150-6. PubMed PMID: 17226054.
38. Eichmann C, Berger B, Reinhold M, Lutz M, Parson W. Canine-specific STR typing of saliva traces on dog bite wounds. Int J Legal Med. 2004;118(6):337-42. doi: 10.1007/s00414-004-0479-7. PubMed PMID: 15480731.
39. Schneider PM, Seo Y, Rittner C. Forensic mtDNA hair analysis excludes a dog from having caused a traffic accident. Int J Legal Med. 1999;112(5):315-6. PubMed PMID: 10460424.

40. Dove CJ, Dahlan NF, Heacker M. Forensic bird-strike identification techniques used in an accident investigation at Wiley Post Airport, Oklahoma, 2008. Human–Wildlife Conflicts. 2009;3(2):179–85.
41. Tobe SS, Reid SJ, Linacre AMT. Successful DNA typing of a drug positive urine sample from a race horse. Forensic Sci Int. 2007;173(1):85-6. doi: 10.1016/j.forsciint.2006.08.009.
42. Liron JP, Ripoli MV, Garcia PP, Giovambattista G. Assignment of paternity in a judicial dispute between two neighbor Holstein dairy farmers. J Forensic Sci. 2004;49(1):96-8. PubMed PMID: 14979351.
43. van de Goor LH, van Haeringen WA, Lenstra JA. Population studies of 17 equine STR for forensic and phylogenetic analysis. Anim Genet. 2011;42(6):627-33. doi: 10.1111/j.1365-2052.2011.02194.x. PubMed PMID: 22035004.
44. Kanthaswamy S, Tom BK, Mattila AM, Johnston E, Dayton M, Kinaga J, et al. Canine population data generated from a multiplex STR kit for use in forensic casework. J Forensic Sci. 2009;54(4):829-40. doi: 10.1111/j.1556-4029.2009.01080.x. PubMed PMID: 19486242.
45. van Asch B, Alves C, Gusmao L, Pereira V, Pereira F, Amorim A. A new autosomal STR nineplex for canine identification and parentage testing. Electrophoresis. 2009;30(2):417-23. doi: 10.1002/elps.200800307. PubMed PMID: 19204943.
46. Ogden R, Mellanby RJ, Clements D, Gow AG, Powell R, McEwing R. Genetic data from 15 STR loci for forensic individual identification and parentage analyses in UK domestic dogs (Canis lupus familiaris). Forensic Sci Int Genet. 2012;6(2):e63-5. doi: 10.1016/j.fsigen.2011.04.015. PubMed PMID: 21600864.
47. Lee JC, Tsai LC, Kuan YY, Chien WH, Chang KT, Wu CH, et al. Racing pigeon identification using STR and chromo-helicase DNA binding gene markers. Electrophoresis. 2007;28(23):4274-81. doi: 10.1002/elps.200700063. PubMed PMID: 18041042.
48. Coghlan ML, White NE, Parkinson L, Haile J, Spencer PB, Bunce M. Egg forensics: an appraisal of DNA sequencing to assist in species identification of illegally smuggled eggs. Forensic Sci Int Genet. 2012;6(2):268-73. doi: 10.1016/j.fsigen.2011.06.006. PubMed PMID: 21741338.
49. Singh A, Gaur A, Shailaja K, Satyare Bala B, Singh L. A novel microsatellite (STR) marker for forensic identification of big cats in India. Forensic Sci Int. 2004;141(2-3):143-7. doi: 10.1016/j.forsciint.2004.01.015. PubMed PMID: 15062954.
50. Wu H, Wan QH, Fang SG, Zhang SY. Application of mitochondrial DNA sequence analysis in the forensic identification of Chinese sika deer subspecies. Forensic Sci Int. 2005;148(2-3):101-5. doi: 10.1016/j.forsciint.2004.04.072. PubMed PMID: 15639603.
51. Kitpipit T, Tobe SS, Kitchener AC, Gill P, Linacre A. The development and validation of a single SNaPshot multiplex for tiger species and subspecies identification--implications for forensic purposes. Forensic Sci Int Genet. 2012;6(2):250-7. doi: 10.1016/j.fsigen.2011.06.001. PubMed PMID: 21723800.
52. Barbanera F, Guerrini M, Beccani C, Forcina G, Anayiotos P, Panayides P. Conservation of endemic and threatened wildlife: molecular forensic DNA against poaching of the Cypriot mouflon (Ovis orientalis ophion, Bovidae). Forensic Sci Int Genet. 2012;6(5):671-5. doi: 10.1016/j.fsigen.2011.12.001. PubMed PMID: 22226984.

53. Lorenzini R, Cabras P, Fanelli R, Carboni GL. Wildlife molecular forensics: identification of the Sardinian mouflon using STR profiling and the Bayesian assignment test. Forensic Sci Int Genet. 2011;5(4):345-9. doi: 10.1016/j.fsigen.2011.01.012. PubMed PMID: 21371958.
54. Lorenzini R. DNA forensics and the poaching of wildlife in Italy: a case study. Forensic Sci Int. 2005;153(2-3):218-21. doi: 10.1016/j.forsciint.2005.04.032. PubMed PMID: 15921870.
55. Nowakowska JA, Oszako T, Tereba A, Konecka A. Forest Tree Species Traced with a DNA-Based Proof for Illegal Logging Case in Poland. In: Pontarotti P, editor. Evolutionary Biology: Biodiversification from Genotype to Phenotype. Cham: Springer International Publishing; 2015. p. 373-88.
56. Nazareno AG, dos Reis MS. Where did they come from? Genetic diversity and forensic investigation of the threatened palm species Butia eriospatha. Conserv Genet. 2014;15(2):441-52. doi: 10.1007/s10592-013-0552-1.
57. Naue J, Lutz-Bonengel S, Pietsch K, Sanger T, Schlauderer N, Schmidt U. Bite through the tent. Int J Legal Med. 2012;126(3):483-8. doi: 10.1007/s00414-012-0674-x. PubMed PMID: 22361950.
58. Caniglia R, Fabbri E, Mastrogiuseppe L, Randi E. Who is who? Identification of livestock predators using forensic genetic approaches. Forensic Sci Int Genet. 2013;7(3):397-404. doi: 10.1016/j.fsigen.2012.11.001. PubMed PMID: 23200859.
59. Dayton M, Koskinen MT, Tom BK, Mattila AM, Johnston E, Halverson J, et al. Developmental validation of short tandem repeat reagent kit for forensic DNA profiling of canine biological material. Croat Med J. 2009;50(3):268-85. PubMed PMID: 19480022; PubMed Central PMCID: PMCPMC2702741.
60. Goncalves J, Pereira F, Amorim A, van Asch B. New method for the simultaneous identification of cow, sheep, goat, and water buffalo in dairy products by analysis of short species-specific mitochondrial DNA targets. J Agric Food Chem. 2012;60(42):10480-5. doi: 10.1021/jf3029896. PubMed PMID: 23025240.
61. Werner FA, Durstewitz G, Habermann FA, Thaller G, Kramer W, Kollers S, et al. Detection and characterization of SNPs useful for identity control and parentage testing in major European dairy breeds. Anim Genet. 2004;35(1):44-9. PubMed PMID: 14731229.
62. Mafra I, Roxo Á, Ferreira IMPLVO, Oliveira MBPP. A duplex polymerase chain reaction for the quantitative detection of cows' milk in goats' milk cheese. Int Dairy J. 2007;17(9):1132-8. doi: http://dx.doi.org/10.1016/j.idairyj.2007.01.009.
63. De S, Brahma B, Polley S, Mukherjee A, Banerjee D, Gohaina M, et al. Simplex and duplex PCR assays for species specific identification of cattle and buffalo milk and cheese. Food Control. 2011;22(5):690-6. doi: http://dx.doi.org/10.1016/j.foodcont.2010.09.026.
64. Doukakis P, Pikitch EK, Rothschild A, DeSalle R, Amato G, Kolokotronis SO. Testing the effectiveness of an international conservation agreement: marketplace forensics and CITES caviar trade regulation. PLoS One. 2012;7(7):e40907. doi: 10.1371/journal.pone.0040907. PubMed PMID: 22848410; PubMed Central PMCID: PMCPMC3405056.
65. DeSalle R, Birstein VJ. PCR identification of black caviar. Nature. 1996;381(6579):197-8.
66. Kumar A, Kumar RR, Sharma BD, Gokulakrishnan P, Mendiratta SK, Sharma D. Identification of species origin of meat and meat products on the DNA basis: a

review. Crit Rev Food Sci Nutr. 2015;55(10):1340-51. doi: 10.1080/10408398.2012.693978. PubMed PMID: 24915324.
67. Wong KL, Wang J, But PP, Shaw PC. Application of cytochrome b DNA sequences for the authentication of endangered snake species. Forensic Sci Int. 2004;139(1):49-55. PubMed PMID: 14687773.
68. Ali ME, Razzak MA, Hamid SB, Rahman MM, Amin MA, Rashid NR, et al. Multiplex PCR assay for the detection of five meat species forbidden in Islamic foods. Food Chem. 2015;177:214-24. doi: 10.1016/j.foodchem.2014.12.098. PubMed PMID: 25660879.
69. Helyar SJ, Lloyd HA, de Bruyn M, Leake J, Bennett N, Carvalho GR. Fish product mislabelling: failings of traceability in the production chain and implications for illegal, unreported and unregulated (IUU) fishing. PLoS One. 2014;9(6):e98691. doi: 10.1371/journal.pone.0098691. PubMed PMID: 24921655; PubMed Central PMCID: PMCPMC4055496.
70. Stern DB, Castro Nallar E, Rathod J, Crandall KA. DNA Barcoding analysis of seafood accuracy in Washington, D.C. restaurants. PeerJ. 2017;5:e3234. doi: 10.7717/peerj.3234. PubMed PMID: 28462038; PubMed Central PMCID: PMCPMC5407275.
71. Bazakos C, Khanfir E, Aoun M, Spano T, Zein ZE, Chalak L, et al. The potential of SNP-based PCR-RFLP capillary electrophoresis analysis to authenticate and detect admixtures of Mediterranean olive oils. Electrophoresis. 2016;37(13):1881-90. doi: 10.1002/elps.201500537. PubMed PMID: 26864388.
72. Pasqualone A, Montemurro C, Caponio F, Blanco A. Identification of virgin olive oil from different cultivars by analysis of DNA microsatellites. J Agric Food Chem. 2004;52(5):1068-71. doi: 10.1021/jf0348424. PubMed PMID: 14995099.
73. Giménez MJ, Pistón F, Martín A, Atienza SG. Application of real-time PCR on the development of molecular markers and to evaluate critical aspects for olive oil authentication. Food Chem. 2010;118(2):482-7. doi: http://dx.doi.org/10.1016/j.foodchem.2009.05.012.
74. Prosser SW, Hebert PD. Rapid identification of the botanical and entomological sources of honey using DNA metabarcoding. Food Chem. 2017;214:183-91. doi: 10.1016/j.foodchem.2016.07.077. PubMed PMID: 27507464.
75. Xanthopoulou A, Ganopoulos I, Kalivas A, Osathanunkul M, Chatzopoulou P, Tsaftaris A, et al. Multiplex HRM analysis as a tool for rapid molecular authentication of nine herbal teas. Food Control. 2016;60:113-6. doi: http://dx.doi.org/10.1016/j.foodcont.2015.07.021.
76. Stoeckle MY, Gamble CC, Kirpekar R, Young G, Ahmed S, Little DP. Commercial teas highlight plant DNA barcode identification successes and obstacles. Sci Rep. 2011;1:42. doi: 10.1038/srep00042. PubMed PMID: 22355561; PubMed Central PMCID: PMCPMC3216529.
77. Ferreira T, Farah A, Oliveira TC, Lima IS, Vitório F, Oliveira EMM. Using Real-Time PCR as a tool for monitoring the authenticity of commercial coffees. Food Chem. 2016;199:433-8. doi: http://dx.doi.org/10.1016/j.foodchem.2015.12.045.
78. Coghlan ML, Haile J, Houston J, Murray DC, White NE, Moolhuijzen P, et al. Deep sequencing of plant and animal DNA contained within traditional Chinese medicines reveals legality issues and health safety concerns. PLoS Genet. 2012;8(4):e1002657. doi: 10.1371/journal.pgen.1002657. PubMed PMID: 22511890; PubMed Central PMCID: PMCPMC3325194.
79. Moon BC, Kim WJ, Ji Y, Lee YM, Kang YM, Choi G. Molecular identification of the traditional herbal medicines, Arisaematis Rhizoma and Pinelliae Tuber, and

common adulterants via universal DNA barcode sequences. Genet Mol Res. 2016;15(1). doi: 10.4238/gmr.15017064. PubMed PMID: 26909979.
80. Galimberti A, De Mattia F, Losa A, Bruni I, Federici S, Casiraghi M, et al. DNA barcoding as a new tool for food traceability. Food Res Int. 2013;50(1):55-63. doi: http://dx.doi.org/10.1016/j.foodres.2012.09.036.
81. Goffaux F, China B, Dams L, Clinquart A, Daube G. Development of a genetic traceability test in pig based on single nucleotide polymorphism detection. Forensic Sci Int. 2005;151(2-3):239-47. doi: 10.1016/j.forsciint.2005.02.013. PubMed PMID: 15939158.
82. Heaton MP, Keen JE, Clawson ML, Harhay GP, Bauer N, Shultz C, et al. Use of bovine single nucleotide polymorphism markers to verify sample tracking in beef processing. J Am Vet Med Assoc. 2005;226(8):1311-4. PubMed PMID: 15844419.
83. Bruni I, Galimberti A, Caridi L, Scaccabarozzi D, De Mattia F, Casiraghi M, et al. A DNA barcoding approach to identify plant species in multiflower honey. Food Chem. 2015;170:308-15. doi: http://dx.doi.org/10.1016/j.foodchem.2014.08.060.
84. Ortola-Vidal A, Schnerr H, Rojmyr M, Lysholm F, Knight A. Quantitative identification of plant genera in food products using PCR and Pyrosequencing® technology. Food Control. 2007;18(8):921-7. doi: http://dx.doi.org/10.1016/j.foodcont.2006.04.013.
85. Ganopoulos I, Argiriou A, Tsaftaris A. Adulterations in Basmati rice detected quantitatively by combined use of microsatellite and fragrance typing with High Resolution Melting (HRM) analysis. Food Chem. 2011;129(2):652-9. doi: http://dx.doi.org/10.1016/j.foodchem.2011.04.109.
86. Johnson CE, Premasuthan A, Satkoski Trask J, Kanthaswamy S. Species identification of Cannabis sativa using real-time quantitative PCR (qPCR). J Forensic Sci. 2013;58(2):486-90. doi: 10.1111/1556-4029.12055. PubMed PMID: 23406349.
87. Dufresnes C, Jan C, Bienert F, Goudet J, Fumagalli L. Broad-Scale Genetic Diversity of Cannabis for Forensic Applications. PLoS One. 2017;12(1):e0170522. doi: 10.1371/journal.pone.0170522. PubMed PMID: 28107530.
88. Congiu L, Chicca M, Cella R, Rossi R, Bernacchia G. The use of random amplified polymorphic DNA (RAPD) markers to identify strawberry varieties: a forensic application. Mol Ecol. 2000;9(2):229-32. PubMed PMID: 10672167.
89. Fernandez ME, Rogberg-Munoz A, Liron JP, Goszczynski DE, Ripoli MV, Carino MH, et al. Effectiveness of single-nucleotide polymorphisms to investigate cattle rustling. J Forensic Sci. 2014;59(6):1607-13. doi: 10.1111/1556-4029.12562. PubMed PMID: 25039316.
90. Beamonte D, Guerra A, Ruiz B, Alemany J. Microsatellite DNA polymorphism analysis in a case of an illegal cattle purchase. J Forensic Sci. 1995;40(4):692-4. PubMed PMID: 7595312.
91. Primmer CR, Koskinen MT, Piironen J. The one that did not get away: individual assignment using microsatellite data detects a case of fishing competition fraud. Proc Biol Sci. 2000;267(1453):1699-704. doi: 10.1098/rspb.2000.1197. PubMed PMID: 11467434; PubMed Central PMCID: PMCPMC1690726.
92. Nielsen EE, Cariani A, Mac Aoidh E, Maes GE, Milano I, Ogden R, et al. Gene-associated markers provide tools for tackling illegal fishing and false eco-certification. Nat Commun. 2012;3:851. doi: 10.1038/ncomms1845. PubMed PMID: 22617291.

93. Grobler JP, Kotze A, Swart H, Hallerman EM. The application of microsatellite DNA markers for forensic analysis of koi carp (Cyprinus carpio). S Afr J Sci. 2005;101:19-21.
94. Gupta SK, Thangaraj K, Singh L. Identification of the source of ivory idol by DNA analysis. J Forensic Sci. 2011;56(5):1343-5. doi: 10.1111/j.1556-4029.2011.01750.x. PubMed PMID: 21392005.
95. Wasser SK, Joseph Clark W, Drori O, Stephen Kisamo E, Mailand C, Mutayoba B, et al. Combating the illegal trade in African elephant ivory with DNA forensics. Conserv Biol. 2008;22(4):1065-71. doi: 10.1111/j.1523-1739.2008.01012.x. PubMed PMID: 18786100.
96. Wasser SK, Mailand C, Booth R, Mutayoba B, Kisamo E, Clark B, et al. Using DNA to track the origin of the largest ivory seizure since the 1989 trade ban. Proc Natl Acad Sci U S A. 2007;104(10):4228-33. doi: 10.1073/pnas.0609714104. PubMed PMID: 17360505; PubMed Central PMCID: PMCPMC1805457.
97. Heaton MP, Leymaster KA, Kalbfleisch TS, Kijas JW, Clarke SM, McEwan J, et al. SNPs for parentage testing and traceability in globally diverse breeds of sheep. PLoS One. 2014;9(4):e94851. doi: 10.1371/journal.pone.0094851. PubMed PMID: 24740156; PubMed Central PMCID: PMCPMC3989260.
98. Heaton MP, Harhay GP, Bennett GL, Stone RT, Grosse WM, Casas E, et al. Selection and use of SNP markers for animal identification and paternity analysis in U.S. beef cattle. Mamm Genome. 2002;13(5):272-81. doi: 10.1007/s00335-001-2146-3. PubMed PMID: 12016516.
99. Rohrer GA, Freking BA, Nonneman D. Single nucleotide polymorphisms for pig identification and parentage exclusion. Anim Genet. 2007;38(3):253-8. doi: 10.1111/j.1365-2052.2007.01593.x. PubMed PMID: 17433014.
100. Himmelberger AL, Spear TF, Satkoski JA, George DA, Garnica WT, Malladi VS, et al. Forensic utility of the mitochondrial hypervariable region 1 of domestic dogs, in conjunction with breed and geographic information. J Forensic Sci. 2008;53(1):81-9. doi: 10.1111/j.1556-4029.2007.00615.x. PubMed PMID: 18279243.
101. Santos S, Oliveira M, Amorim A, van Asch B. A forensic perspective on the genetic identification of grapevine (Vitis vinifera L.) varieties using STR markers. Electrophoresis. 2014;35(21-22):3201-7. doi: 10.1002/elps.201400107. PubMed PMID: 25146979.
102. Pereira L, Gomes S, Castro C, Eiras-Dias JE, Brazao J, Graca A, et al. High Resolution Melting (HRM) applied to wine authenticity. Food Chem. 2017;216:80-6. doi: 10.1016/j.foodchem.2016.07.185. PubMed PMID: 27596395.
103. Grantham NS, Reich BJ, Pacifici K, Laber EB, Menninger HL, Henley JB, et al. Fungi identify the geographic origin of dust samples. PLoS One. 2015;10(4):e0122605. doi: 10.1371/journal.pone.0122605. PubMed PMID: 25875229; PubMed Central PMCID: PMCPMC4395444.
104. Malewski T, Draber-Monko A, Pomorski J, Los M, Bogdanowicz W. Identification of forensically important blowfly species (Diptera: Calliphoridae) by high-resolution melting PCR analysis. Int J Legal Med. 2010;124(4):277-85. doi: 10.1007/s00414-009-0396-x. PubMed PMID: 20082091.
105. Boykin LM, Armstrong KF, Kubatko L, De Barro P. Species delimitation and global biosecurity. Evol Bioinform Online. 2012;8:1-37. doi: 10.4137/EBO.S8532. PubMed PMID: 22267902; PubMed Central PMCID: PMCPMC3256992.

106. Köppel R, Ganeshan A, van Velsen F, Bucher T. Five pentaplex real-time PCR systems for the efficient determination of 20 genetically modified maize traits in food. Eur Food Res Technol. 2017;243(2):215-25. doi: 10.1007/s00217-016-2737-6.
